# Leydig cell tumor of a testis with azoospermia: A case report and literature review

**DOI:** 10.1097/MD.0000000000022085

**Published:** 2020-09-04

**Authors:** Qingkuo Kong, Yang Yu, Tian Tian, Hongguo Zhang, Meiling Sun, Ruizhi Liu, Yanhong Liu

**Affiliations:** Center for Reproductive Medicine, Center for Prenatal Diagnosis, First Hospital, Jilin University, Changchun, Jilin 130021, PR China.

**Keywords:** azoospermia, hormone, Leydig cell tumors, natural pregnancy

## Abstract

**Rationale:**

Testicular tumors represent 1% to 1.5% of all tumors in men. Those derived from Leydig cells are rare and account for 1% of testicular tumors. Leydig tumor cells can produce steroid hormones such as estrogen, progesterone and testosterone. The amount and type of hormones secreted by these tumors may produce complicated clinical characteristics in these patients.

**Patient concerns:**

Here, we report a patient with azoospermia, a testicular Leydig cell tumor (LCT), and elevated plasma testosterone levels. We describe the diagnostic and therapeutic experience of this case, and our follow-up of the patient's clinical indicators and fertility status.

**Diagnosis:**

The patient was diagnosed with azoospermia and a testicular LCT.

**Interventions:**

The patient underwent testicular tumor removal and long-term follow-up.

**Outcomes:**

After 4 months of follow-up, the patient's semen examination index significantly improved and his wife became naturally pregnant. At 4 months of gestation, the fetus was delivered because of a ruptured amniotic cavity. Twenty-six months after tumor removal, the patient's sex hormone levels had completely returned to normal and spermatogenic function had partially recovered, but there was no natural pregnancy with his partner.

**Conclusion:**

For LCTs, testis sparing surgery may provide a safe and feasible option to restore spermatogenic function, although longer-term follow-up is required. Drug assistance may be required to maintain spermatogenic function and achieve fertility, and further research is required.

## 1 Introduction

Testicular tumors account for 1% to 1.5% of all tumors in men, and Leydig cell tumors (LCTs) account for only 1% of total testicular tumors.^[1]^ LCTs are sex cord stromal tumors derived from Leydig cells and generally occur unilaterally, with only 3% of cases found bilaterally.^[2]^ Testicular solid tumors are almost malignant.^[3]^ LCT is a rare, mostly benign tumor in adults, although 10% to 20% may be malignant.^[4]^ Therefore, the identification of LCT and other malignant testicular tumors is important for guiding clinical treatments.

Approximately 20% of LCTs occur in children aged 6 to 10 years, and 80% occur in adults aged 20 to 60 years. The clinical characteristics and hormone levels associated with LCTs vary greatly.^[1,5,6]^ Children may exhibit precocious puberty with symptoms such feminization (10% of patients may display breast development), genital enlargement, appearance of pubic hair, and deepening voice. Adult patients with LCT are mainly treated for a testicular mass or infertility. About 30% have symptoms of feminization, of which male breast development is the most common, and decreased sexual desire, erectile dysfunction, infertility and testicular and prostate atrophy. The different clinical symptoms may be related to the following mechanisms:

(1) steroidogenic tumor cells produce estradiol (E2), progesterone and testosterone (T), and the amount and type of secreted hormones may affect the clinical characteristics of patients;
(2) the activity of aromatase is low in childhood and high in adults. Different levels of aromatase activity will affect the conversion of T to E2 and cause different clinical features.^[1,5,6]^

In addition to the above clinical manifestations, the diagnosis of LCT should also be performed with auxiliary tests such as hormone levels, tumor markers, B-ultrasound, and magnetic resonance (MR). Some LCTs secrete T and/or E2, but tumor markers such as alpha-fetoprotein (AFP), human chorionic gonadotropin (hCG) and lactic acid dehydrogenase (LDH) are often negative. B-ultrasound and MR can diagnose the tumor but cannot determine if it is benign or malignant. Clear diagnosis before surgery is very difficult and diagnosis still largely depends on postoperative pathological examination. LCTs are not sensitive to radiotherapy and chemotherapy, so surgical resection is currently the only effective treatment.^[7,8]^ The present surgical methods mainly include radical orchiectomy and testis sparing surgery (TSS), but the use of these techniques remains controversial.^[1]^ In recent years, several studies have confirmed the safety and effectiveness of TSS for adult LCTs.^[7,9–11]^ For patients with benign tumors, especially children, and for non-fertility, and bilateral testicular disease, TSS surgery is recommended according to rapid intraoperative pathological results. Most patients return to normal after surgery, but some cases require adjuvant medication for fertility.^[12]^ Therefore, we retrospectively analyzed a case of LCT exhibiting azoospermia and elevated plasma T levels and present here the diagnostic and therapeutic experience.

## 2 Case report

The study protocol was approved by the Ethics Committee of the First Hospital of Jilin University (No. 2019-239). Written informed consent was obtained from all patients.

The patient was a 27-year-old married man who came to the Reproductive Medicine Center of the First Clinical Hospital of Jilin University for infertility treatment. The patient had experienced a right testicular bulge accompanied by tenderness, but no loss of sexual desire and erectile dysfunction. Physical examination revealed male appearance, body hair and no male breast development. The penis had developed normally without foreskin phimosis. When standing, the right scrotum drooped and the testicle was heavy with an approximate volume of 20 mL. Tenderness was positive, and the light transmission test was negative. The left testicle was about 8 mL, and there were no varicocele or other abnormalities. The bilateral groin did not touch the enlarged lymph nodes. A routine semen test indicated azoospermia. Hormone measurement results were: follicle-stimulating hormone (FSH): 0.11 (1.50–12.40) mIU/mL, luteinizing hormone (LH): <0.10 (1.70–8.60) mIU/mL, E2: 47.8 (25.8–60.7) pg/mL, prolactin: 217.2 (86.0–324.0) μIU/mL, T: 28.72 (9.90–27.80) nmol/L, LDH: 162 (135–226) U/L, AFP: 0.64 (<7.0) ng/mL, hCG: 2.39 mIU/mL. Testicular ultrasound showed the left testicular size was 34 × 22 × 16 mm (8.5 mL), the right testicular size was 46 × 28 × 21 mm (19.2 mL), and the upper end of the testicle was found in the 24 × 15 mm mixed echo zone, where blood flow signals were observed. MR showed the testicular lesions size to be about 23 × 19 mm, and the short T2 signal was abnormal, the enhanced scan of the coronal lesion was markedly high, and the lesion was adjacent to the epididymis. The prostate capsule was intact and no obvious abnormalities were found in the seminal vesicles, bladder or rectum. No abnormal enlarged lymph nodes were found in the pelvis. No signs of destruction were seen in the bone. There were no detected abnormalities in chromosomes and azoospermia factor region of chromosome Y.

The patient underwent TSS at the First Hospital of Jilin University on May 26, 2017. Pathological examination showed a benign stromal cell tumor with a volume of 30 × 25 × 18 mm (9.6 mL). Seminiferous tubules were found in the tumor, tumor atypia was not obvious, no necrosis or pathological mitosis was observed, the mitotic count was 1/10 high power fields, and no tumor infiltration was observed in the peripheral margin. Immunohistochemical results were: Calretinin (+), Ki-67 (+<1%), ASLL4 (−), Vimentin (+), CK-pan (−), a-inhibin (+).

The patient's wife became naturally pregnant in the 4th month after TSS. The wife was induced at 4 months of pregnancy because of a ruptured amniotic cavity. In September 2018, the patient returned to our hospital after 3 months of infertility. The patient's semen analysis is shown in Table 1. On April 27, 2019, semen examination showed a large fluctuation in sperm density and activity than before, and no treatment was given. In May 2019, the patient received hormone therapy (25 mg Clomiphene, orally once a day), and after 2 months the sperm density and motility and reproductive hormone levels were normal. The specific monitoring indicators are shown in Tables 1 and 2.

**Table 1:**
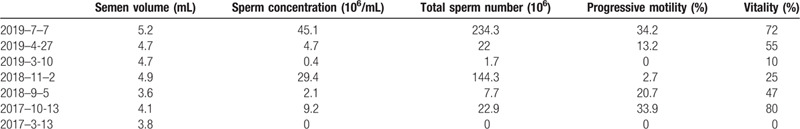
Semen analysis before and after surgery.

**Table 2:**
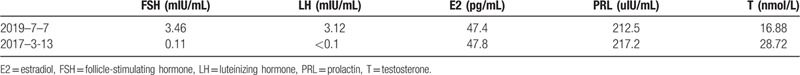
Sex hormone levels before and after surgery.

## 3 Discussion

LCTs secrete androgens, which can be converted to E2 in adults, and the increased androgen and E2 levels exert different clinical manifestations.^[1,5,6]^ In this case, the tumor had an androgen secreting function, and the clinical findings were azoospermia and infertility. After TSS, the postoperative hormone levels returned to normal and spermatogenic function was partially restored. This indicates that the normal secretion of gonadotropins plays an important role in maintaining spermatogenic function. This clinical endpoints was consistent with other case reports of LCTs. Androgen secretion by LCTs was not sufficient to maintain spermatogenesis, likely reflecting a significant androgen-induced decrease in gonadotropin secretion. Sperm production is a very complex physiological process with multiple factors required for male fertility. It is well established that FSH, LH and T must function in an orderly manner during spermatogenesis. High levels of intratesticular T are essential for sperm production. The hypothalamic secretion of gonadotropin-releasing hormone (GnRH) stimulates the pituitary secretion of FSH and LH. LH promotes the secretion of T from Leydig cells, and FSH acts in conjunction with T to promote spermatogenesis via the testicular support cells. Testosterone, E2, dihydrotestosterone, and inhibin B secreted by supporting cells can negatively regulate GnRH, FSH and LH. Elevated T associated with LCT may be aromatized to form E2. In the current case, the patient had elevated plasma T levels and circulating E2 was normal, but FSH and LH serum levels were inhibited, which led to spermatogenic disruption. Therefore, the fluctuation of the postoperative semen index may be related to unstable hormone levels; however, further detailed analysis of hormone levels in the postoperative period is required.

The main treatment for LCT is orchiectomy. The European Urological Association guidelines state that TSS is not recommended for unilateral testicular tumors, but TSS can be performed in certain special cases such as prepubertal or fertile patients. However, even if a postoperative pathological diagnosis indicates a benign tumor, regular follow-up is required. Gianluca et al^[7]^ reported that for 17 patients that underwent TSS, the pathology showed no marked signs of malignancy, except for a slight increase in mitotic figures in tumors with a diameter of *31 mm*, and no recurrence or metastasis was observed after *12–192* months of follow-up. Nazareno et al^[9]^ reported that 29 of 37 patients with LCT underwent TSS. The median follow-up time was 4.6 years, and no tumor recurrence or metastasis was observed. Andreas et al^[10]^ compared the therapeutic effects of testicular radical resection and TSS (eight patients). The average tumor diameters for patients with radical resection and TSS were *12.9 mm* and *8.6 mm*, respectively, and the median follow-ups were 77 months and 42 months, respectively. The authors proposed that TSS for LCT was safe. Additionally, Giorgio^[11]^ and other investigators have reported no tumor recurrence after surgery for LCT.

The follow-up treatment for these patients should include examination of fertility, monitoring hormone levels and semen indicators, pregnancy test of partner at 3 months, partner examination (if no pregnancy), and B-ultrasound analysis.

## 4 Conclusion

Hormone-releasing LCTs produce various clinical features. Azoospermia and the diagnosis of LCT have rarely been reported worldwide. In this article, the patient was diagnosed with azoospermia after a year of infertility with a normal sexual life. During diagnosis and treatment, a right testicular mass was found on B-ultrasound. After the testicular tumor was removed, sex hormone levels returned to normal, spermatogenic function was partially restored, and one initial pregnancy was achieved. Prior to the pathological diagnosis of LCT, the detection of plasma sex hormone levels and contrast-enhanced MR scans may facilitate the diagnosis of LCT from other testicular malignancies and guide the choice of surgical methods for patients with LCT. The main treatment for LCT is orchiectomy. For patients with small tumor volumes, prepubertal and bilateral testicular stromal tumors, or for other practical reasons, TSS may provide a safe and feasible treatment, and also restore some spermatogenic function. Drug assistance may be required to maintain spermatogenic function and achieve fertility, and further long-term research is required.

## Author contributions

**Conceptualization:** Yang Yu.

**Formal analysis:** Qingkuo Kong.

**Resources:** Yang Yu.

**Validation:** Hongguo Zhang.

**Writing – original draft:** Qingkuo Kong.

**Writing – review & editing:** Tian Tian, Meiling Sun, Ruizhi Liu.

## References

[1] Al-Agha OM Axiotis CA An in-depth look at Leydig cell tumor of the testis. Arch Pathol Lab Med 2007;131:311 7. 17284120 10.5858/2007-131-311-AILALC

[2] Kim I Young RH Scully RE Leydig cell tumors of the testis. A clinicopathological analysis of 40 cases and review of the literature. Am J Surg Pathol 1985;9:177 92. 3993830 10.1097/00000478-198503000-00002

[3] Mcglynn KA Trabert B Adolescent and adult risk factors for testicular cancer. Nat Rev Urol 2012;9:339 49. 22508459 10.1038/nrurol.2012.61 PMC4031676

[4] Maizlin ZV Belenky A Kunichezky M Leydig cell tumors of the testis: gray scale and color Doppler sonographic appearance. J Ultrasound Med 2004;23:959 64. 15292565 10.7863/jum.2004.23.7.959

[5] Young RH Testicular tumors – some new and a few perennial problems. Arch Pathol Lab Med 2008;132:548 64. 18384207 10.5858/2008-132-548-TTNAAF

[6] Agarwal PK Palmer JS Testicular and paratesticular neoplasms in prepubertal males. J Urol 2006;176:875 81. 16890643 10.1016/j.juro.2006.04.021

[7] Giannarini G Mogorovich A Menchini Fabris F Long-term followup after elective testis sparing surgery for Leydig cell tumors: a single center experience. J Urol 2007;178 (3 Pt 1):872 6. quiz 1129. 17631320 10.1016/j.juro.2007.05.077

[8] Carmignani L Salvioni R Gadda F Long-term followup and clinical characteristics of testicular Leydig cell tumor: experience with 24 cases. J Urol 2006;176:2040 3. discussion 2043. 17070249 10.1016/j.juro.2006.07.005

[9] Suardi N Strada E Colombo R Leydig cell tumour of the testis: presentation, therapy, long-term follow-up and the role of organ-sparing surgery in a single-institution experience. BJU Int 2009;103:197 200. 18990169 10.1111/j.1464-410X.2008.08016.x

[10] Loeser A Vergho DC Katzenberger T Testis-sparing surgery versus radical orchiectomy in patients with Leydig cell tumors. Urology 2009;74:370 2. 19646624 10.1016/j.urology.2009.03.014

[11] Bozzini G Picozzi S Gadda F Long-term follow-up using testicle-sparing surgery for Leydig cell tumor. Clin Genitourin Cancer 2013;11:321 4. 23317518 10.1016/j.clgc.2012.12.008

[12] Hibi H Yamashita K Sumitomo M Leydig cell tumor of the testis, presenting with azoospermia. Reprod Med Biol 2017;16:392 5. 29259494 10.1002/rmb2.12046 PMC5715892

